# Supplementary material for: A cleaner snow future mitigates Northern Hemisphere snowpack loss from warming
Source: Nat Commun. 2023 Oct 2;14:6074. doi: 10.1038/s41467-023-41732-6 (PMC10545800; doi:10.1038/s41467-023-41732-6)
Supplement: Supplementary file 1 — Supplementary Information [file 41467_2023_41732_MOESM1_ESM.pdf]

Supplementary information of

A cleaner snow future mitigates Northern Hemisphere snowpack loss from warming

Dalei Hao[1*], Gautam Bisht[1], Hailong Wang[1], Donghui Xu[1], Huilin Huang[1], Yun Qian[1] and L. Ruby Leung[1*]

[1]Atmospheric Sciences and Global Change Division, Pacific Northwest National Laboratory, Richland, WA, USA

This PDF file contains:

Supplementary Text S1

Supplementary Figures S1-S17

Supplementary Tables S1-S3

Supplementary Text S1

Data processing in the model-observation comparison

We excluded those snow samples affected by drifting snow or with poor spatial representativeness reported in the studies. For the observations estimated from the Integrating Sphere integrating SandWich spectrophotometer, we scaled the measured black carbon (BC) concentration in snow (Supplementary Data 1) to match the BC mass absorption efficiency of 7.5 m^2 g^{-1} used in Energy Exascale Earth System Model (E3SM) Land Model (ELM). We used both the BC concentration in the top snow layer and the snow column. For the snow samples with unreported BC concentration in the snow column, we averaged the surface and sub-surface BC concentration to get the approximate values.

It should be noted that using point-scale field measurements for evaluation of model simulations at a relatively coarse resolution (0.5°) is rather challenging because of the scale mismatch[1] and the strong dependence of snowpack on local topography and microclimate conditions. For the evaluation of historical simulations (before 2015), all snow samples within the same model grid cell for the same month and year were aggregated, and then the observations and simulations were paired according to their specific geo-location, month and year. To utilize the snowpit data for model evaluation, we compared snowpit measurements over the TP available after 2014 with the recent climatological (2005-2014) average of the ELM historical simulations because the ELM simulations after 2014 are driven by projected scenarios rather than the historically observed forcing. Furthermore, we compared the limited snowpit snow samples acquired during summer with the ELM simulated spring average, considering that summer monthly outputs from the ELM simulations show little snow cover during that period.

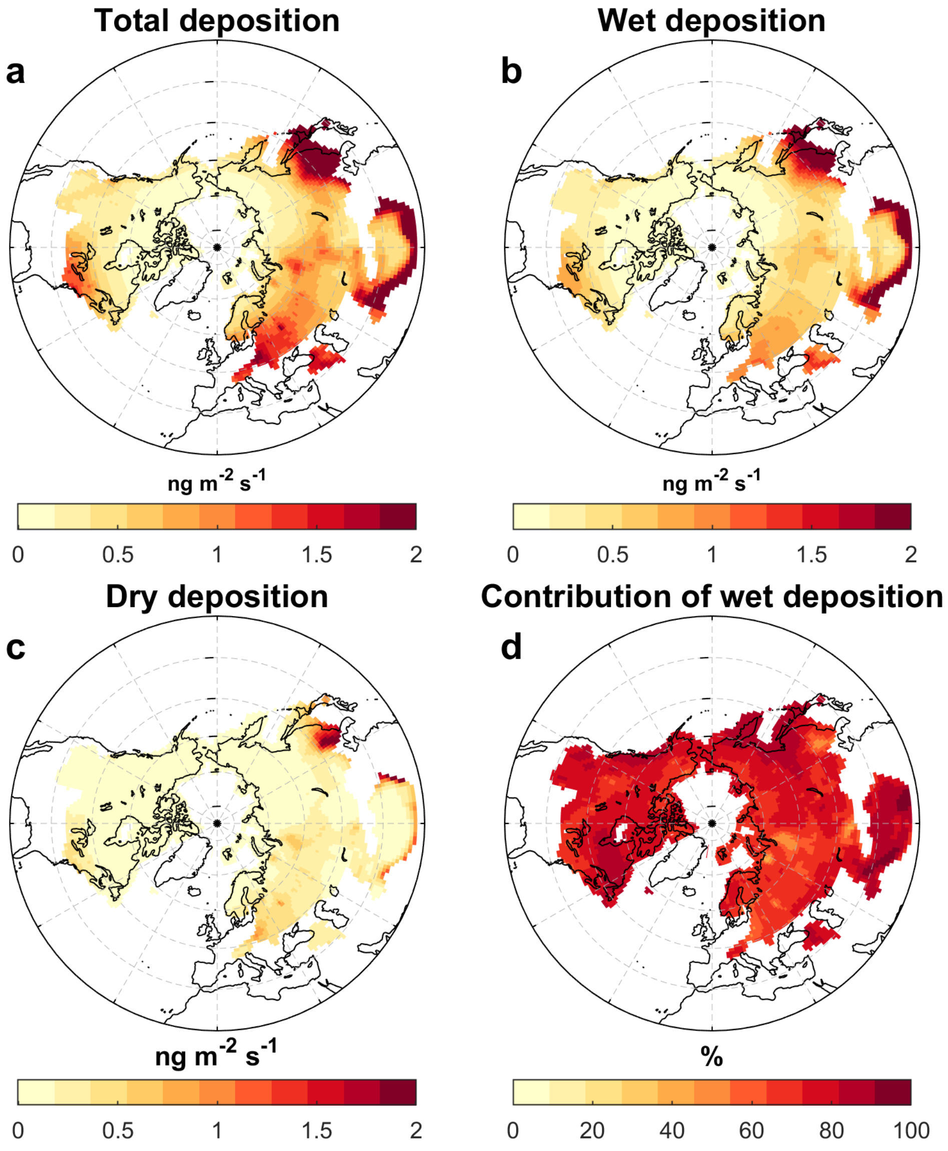

Figure S1| Spatial patterns of historical black carbon (BC) deposition rate over the Northern Hemisphere (NH): (a) total, **(b)** wet, **(c)** dry deposition, and **(d)** the ratio of wet to total deposition. Here, the BC deposition rates are calculated based on the ensemble mean of seven CMIP6 model outputs from December to May. In each panel, grids with an average snow water equivalent (SWE) from December to May smaller than 5 mm are masked.

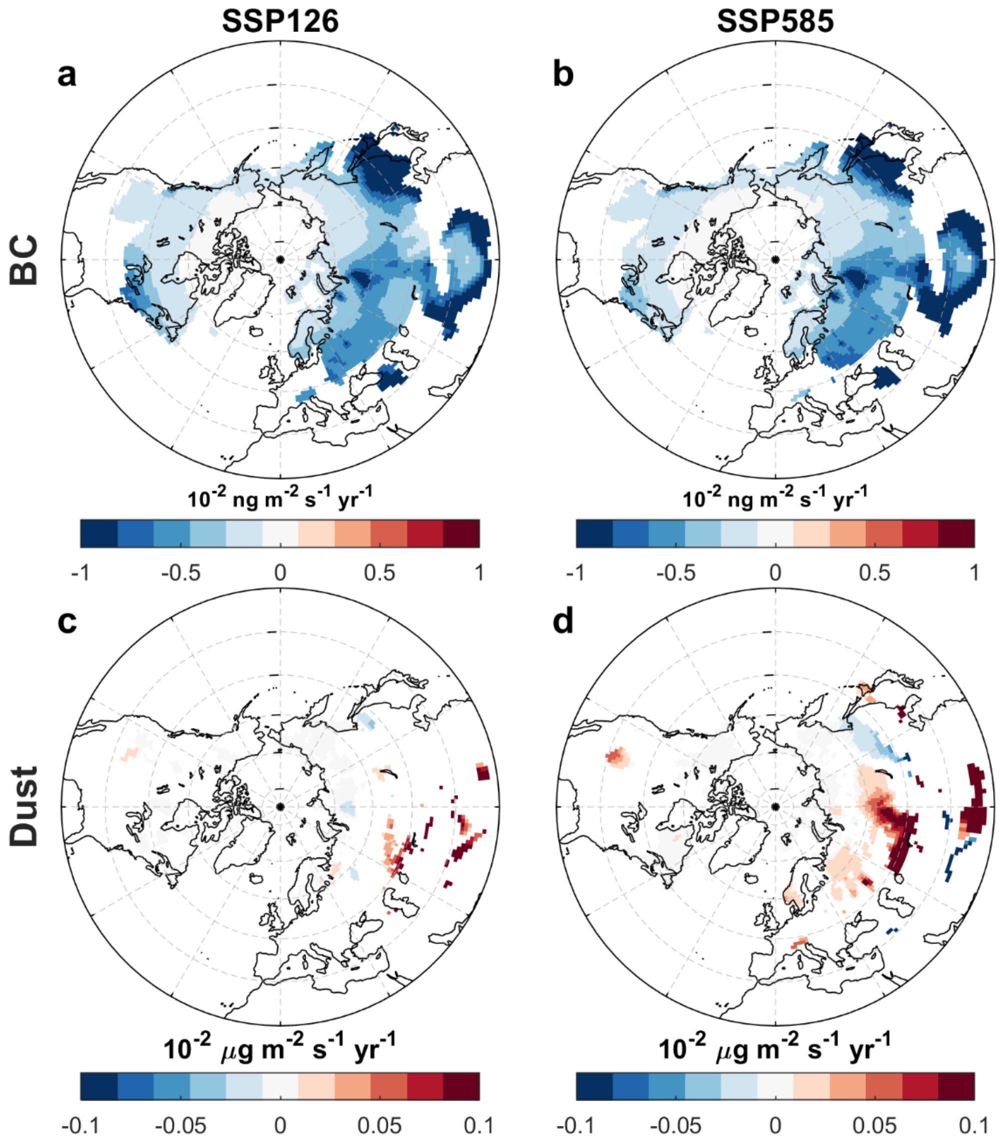

Figure S2| Future trends of black carbon (BC) and dust deposition from 2015-2100 over the Northern Hemisphere (NH)**. a,b** Spatial pattern of the Sen's slopes of BC deposition under SSP126 and SSP585. **c,d** Spatial pattern of the Sen's slopes of dust deposition under SSP126 and SSP585**.** Here, the ensemble mean of seven CMIP6 model outputs from December to May is used to calculate the Sen's slope. In each panel, grids without significant increasing or decreasing trends ($p>0.05$ in the Mann-Kendall (MK) test) or where snow water equivalent (SWE) is smaller than 5 mm are masked.

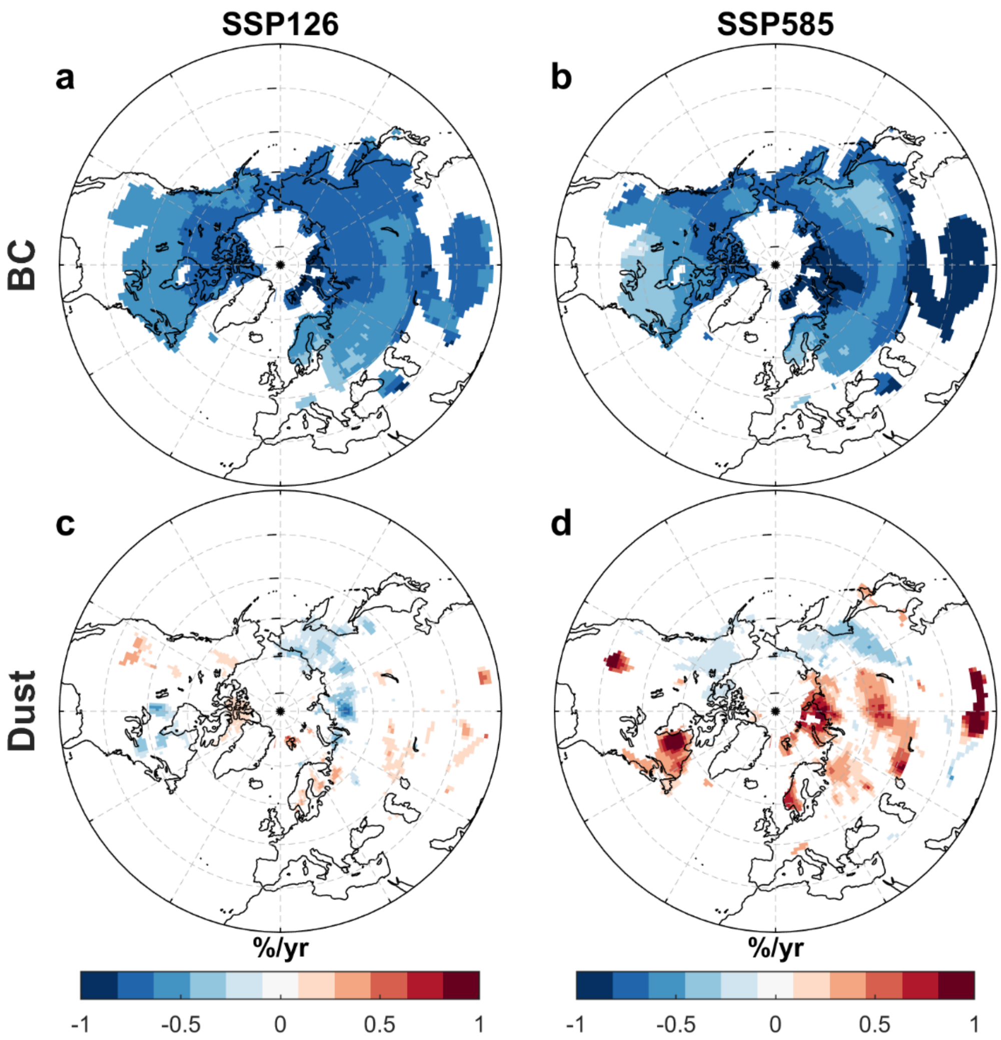

Figure S3| Future relative trends of black carbon (BC) and dust deposition from 2015-2100 over the Northern Hemisphere (NH)**. a,b** Spatial pattern of the relative Sen's slopes of BC deposition under SSP126 and SSP585. **c,d** Spatial pattern of the relative Sen's slopes of dust deposition under SSP126 and SSP585**.** Here, the ensemble mean of seven CMIP6 model outputs from December to May is used to calculate the relative Sen's slope. The relative Sen's slope is calculated as the ratio of the Sen's slope to the historical (1995-2015) average deposition rates. In each panel, grids without significant increasing or decreasing trends ($p>0.05$ in the Mann-Kendall (MK) test) or where snow water equivalent (SWE) is smaller than 5 mm are masked.

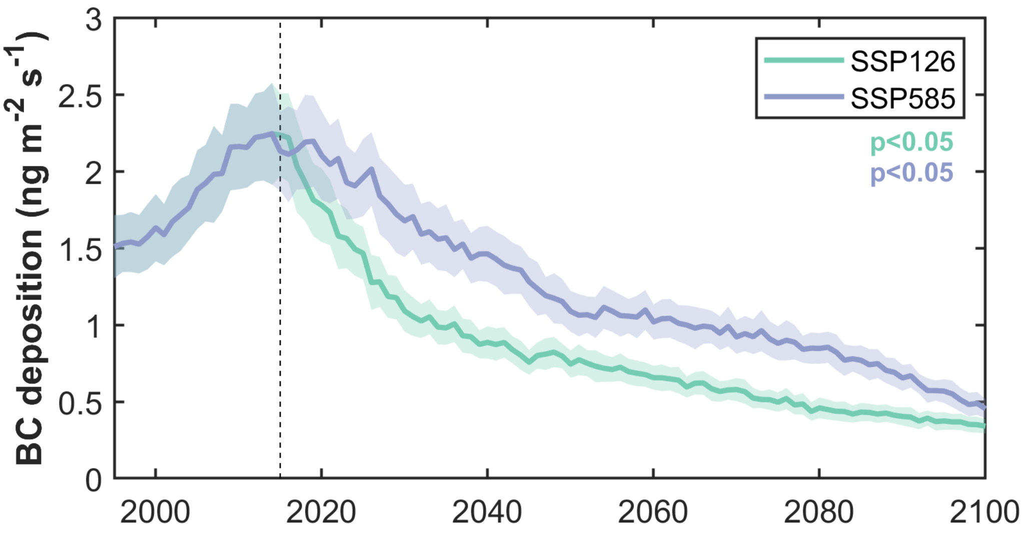

Figure S4| Time series of average black carbon (BC) deposition from December to May over snow-covered Tibetan Plateau (TP) regions (where the average snow water equivalent (SWE) exceeds 5 mm in the historical period of 1995-2014) under SSP126 and SSP585. For each panel, the solid line and background shading represent the mean and standard deviation of BC deposition rates, respectively, based on the seven CMIP6 models. The p values from the Mann-Kendall (MK) test of statistical significance of the temporal trends from 2015-2100 are shown inside each panel.

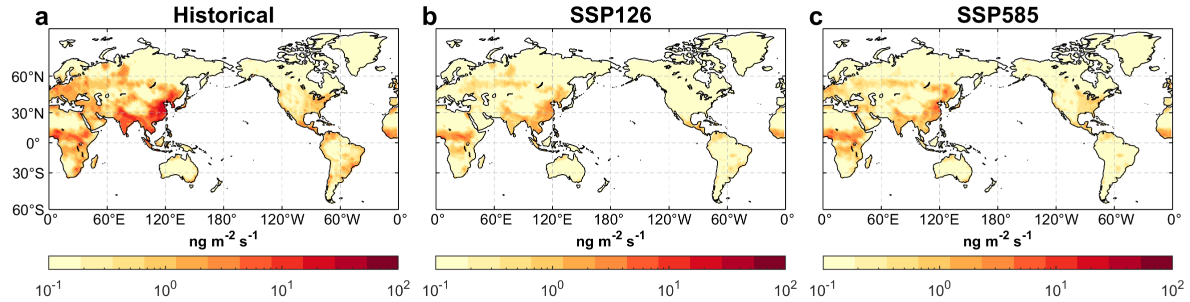

Figure S5| Spatial patterns of historical (1995-2100) and future (2081-2100) black carbon (BC) emission rates over the globe: (**a)** historical, (**b)** SSP126, and (**c)** SSP585. Here, the BC emission rates are calculated based on the ensemble mean of seven CMIP6 model outputs from December to May.

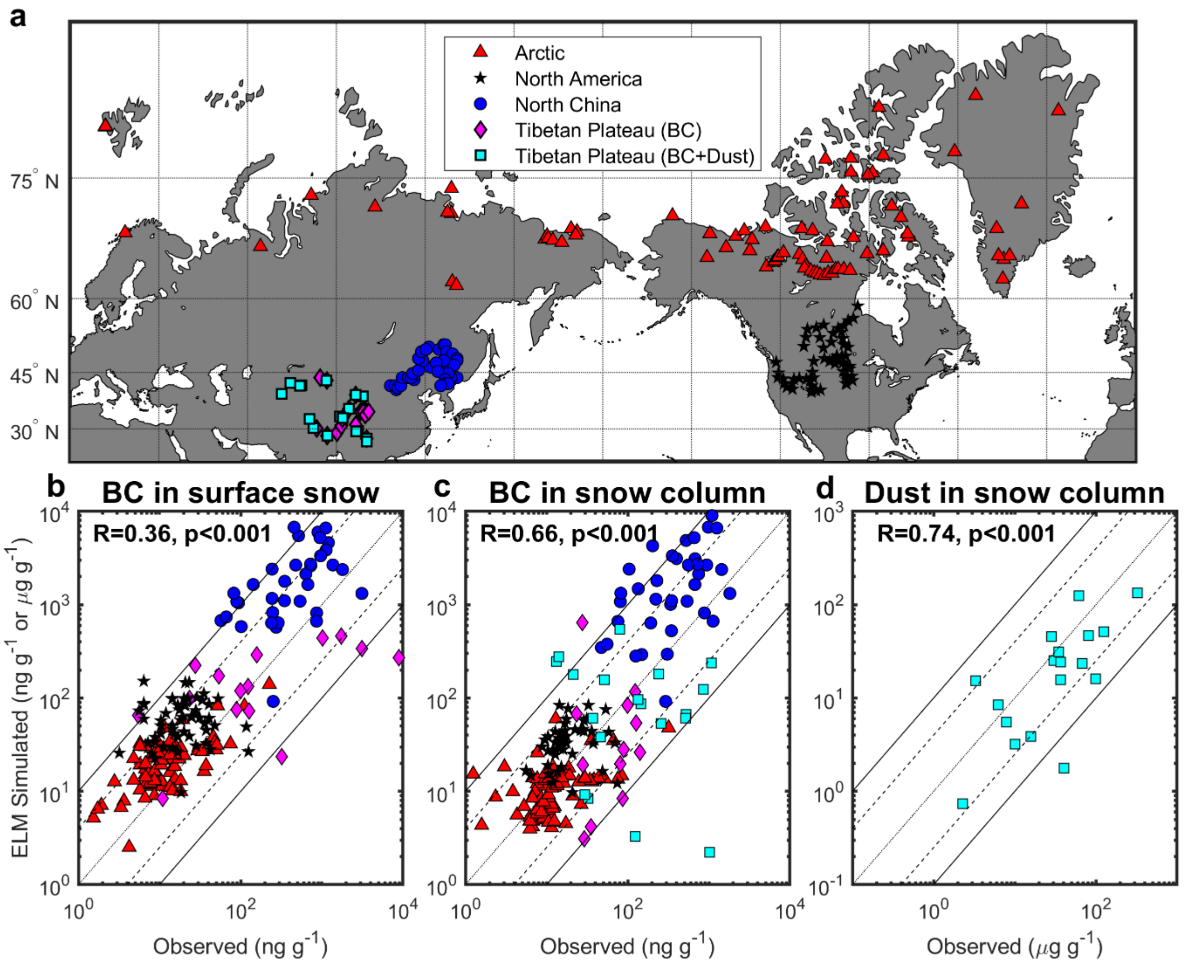

Figure S6| Comparison of ELM-simulated and observed black carbon (BC) and dust concentration in snow across the Northern Hemisphere (NH)**. a.** Spatial distribution of field snow samples. **b,c** Scatter plots between observed and simulated BC concentration in the top snow layer and snow column. **d.** Scatter plots between observed and simulated dust concentration in the snow column. In (**b,c,d**), the dotted, dashed and solid lines are 1:1,f 1:4 (or 4:1) and 1:10 (or 10:1) ratio lines, and the correlation coefficient and p value are labeled.

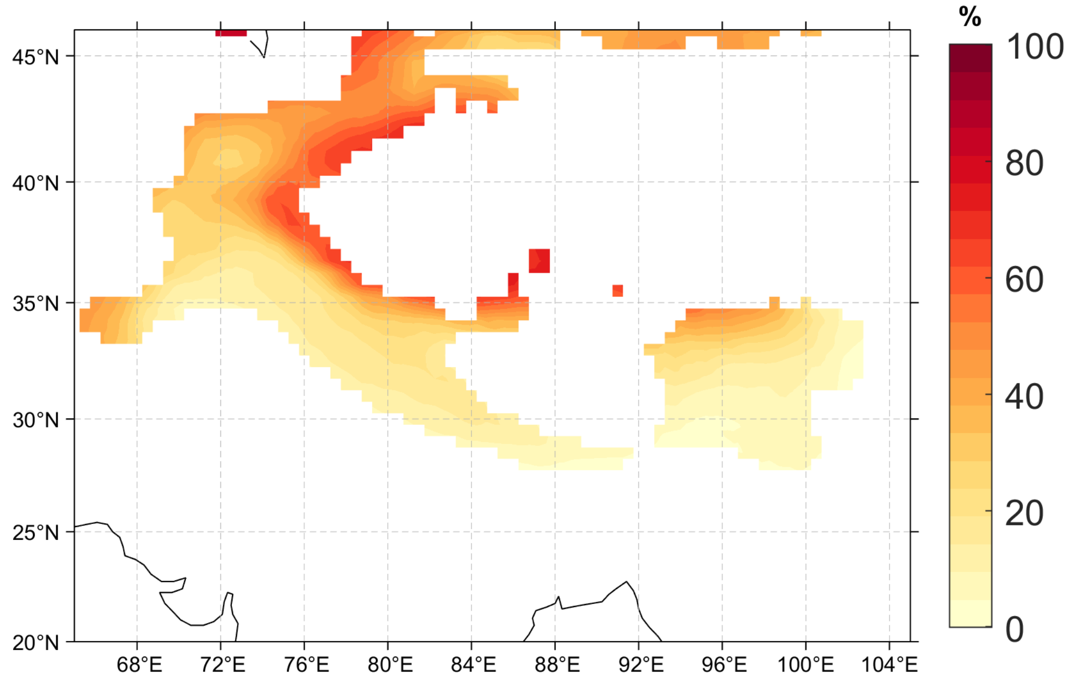

Figure S7| Ratio of historical (1995-2014) average dust-induced albedo reduction to total albedo reduction over the Tibetan Plateau (TP). The snow albedo reduction is calculated based on ELM outputs from December to May. The grids where the average snow water equivalent (SWE) during December to May is smaller than 5 mm are masked.

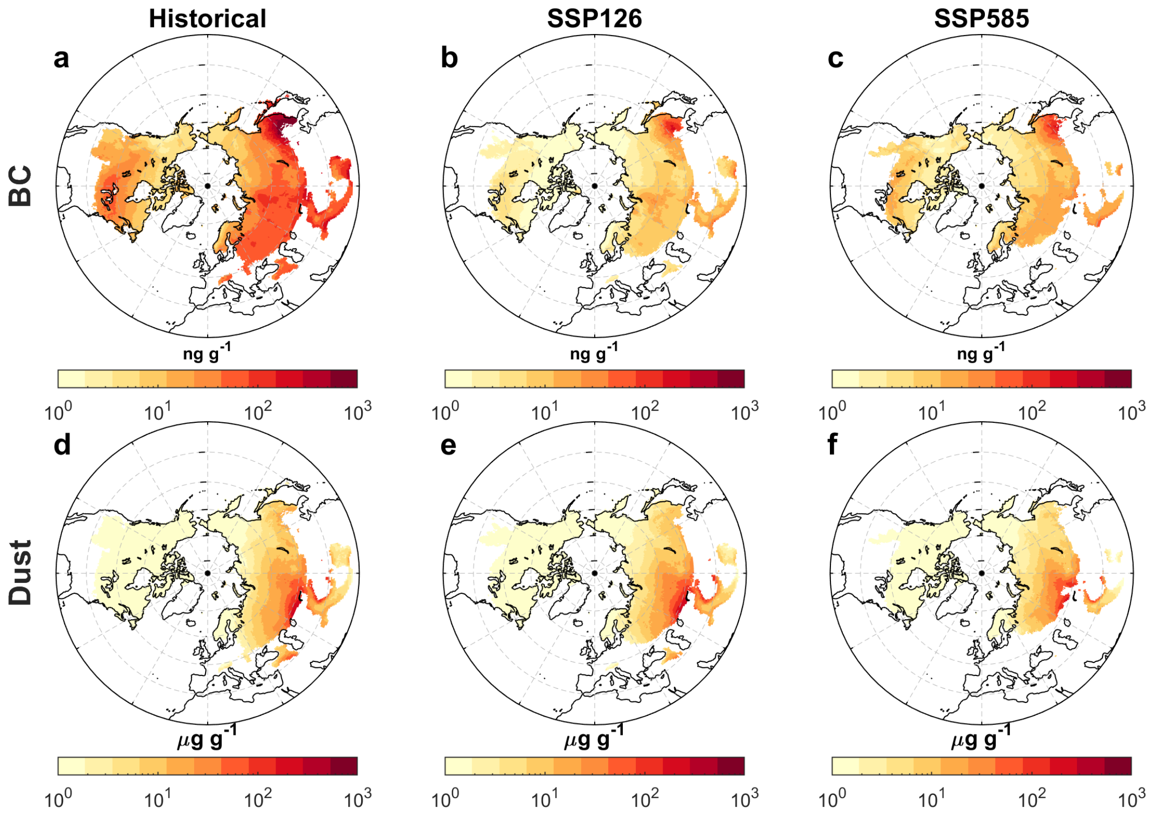

Figure S8| Spatial patterns of historical and future concentrations of black carbon (BC) and dust in snow column over the Northern Hemisphere (NH)**. a,d** Historical (1995-2014) and **b,c,e,f** future (2081-2100) spatial patterns of the concentration of BC and dust in snow column under SSP126 and SSP585. Historical and future light-absorbing particles (LAP) concentrations in snow column are calculated based on ELM outputs for December to May. In each panel, the grids where snow water equivalent (SWE) is smaller than 5 mm are masked.

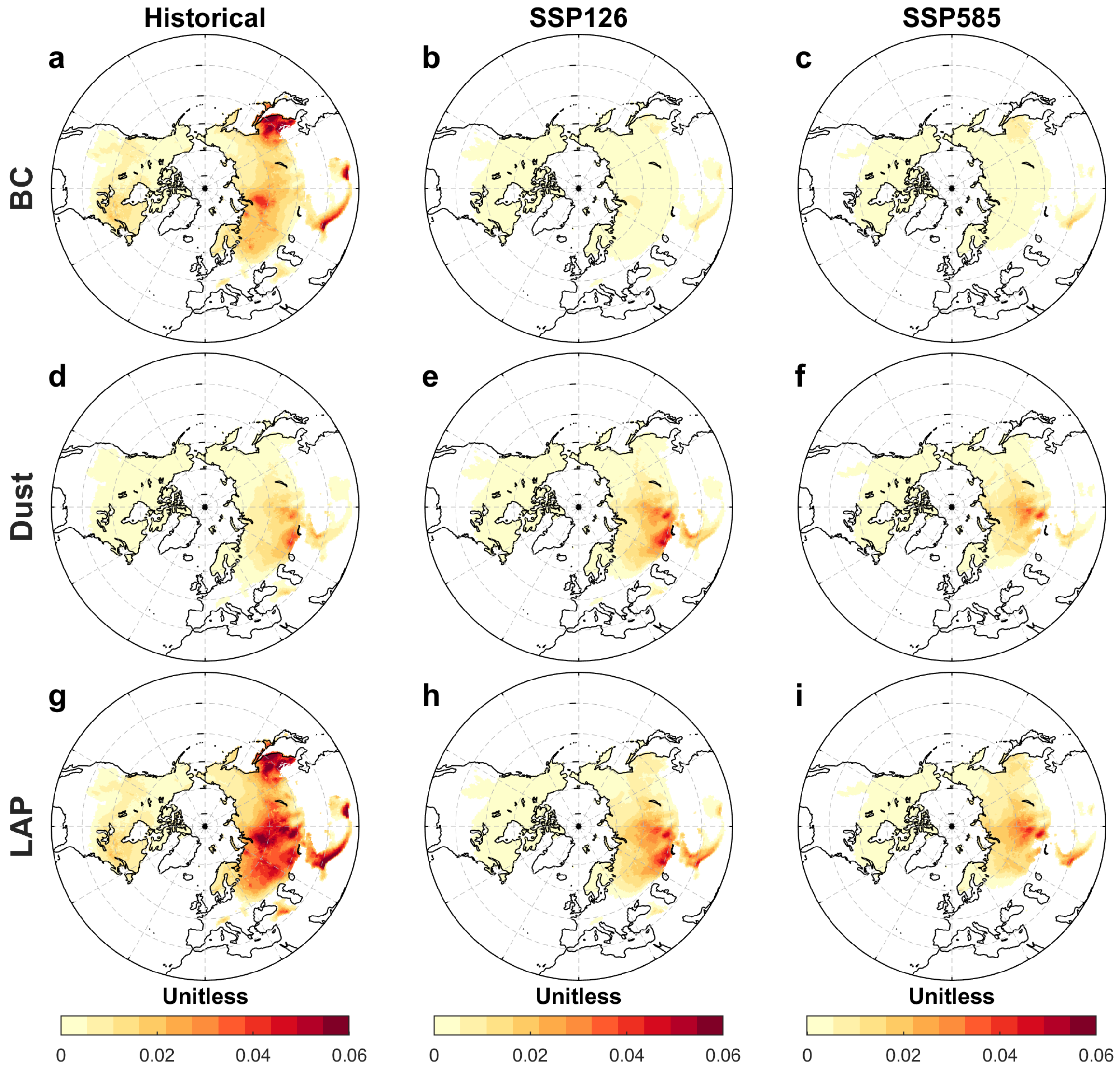

Figure S9| Spatial patterns of historical and future snow albedo reduction caused by black carbon (BC), dust, and light-absorbing particles (LAP) **(the sum of BC and dust) over the** Northern Hemisphere (NH)**. a,d,g** Historical (1995-2014) and **b,c,e,f,h,i** future (2081-2100) spatial patterns of snow albedo reduction under SSP126 and SSP585. Historical and future snow albedo reductions are calculated based on ELM outputs for December to May. In each panel, grids where snow water equivalent (SWE) is smaller than 5 mm are masked.

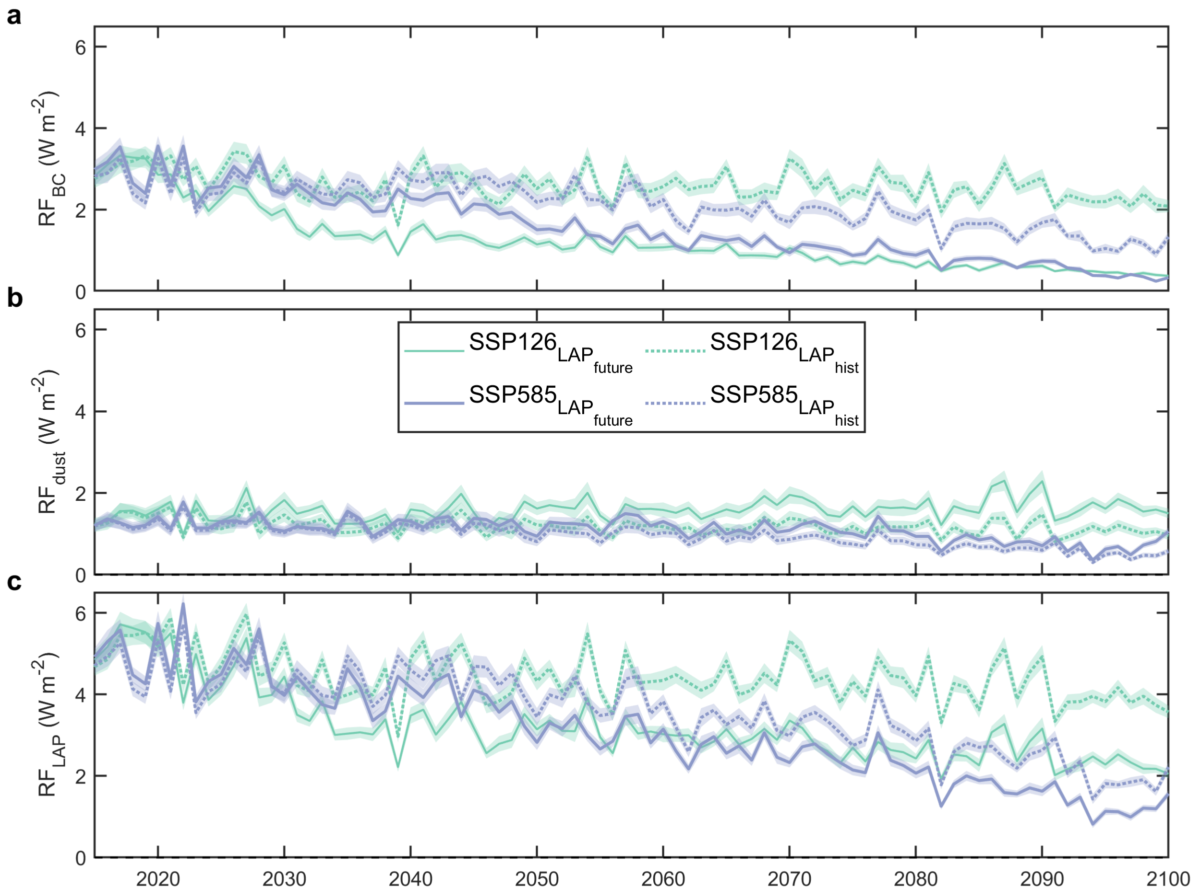

Figure S10| Time series of average surface radiative forcings (RF) from December to May caused by (a) BC, (b) dust, and (c) light-absorbing particles (LAP) (the sum of BC and dust) over snow-covered Tibetan Plateau (TP) regions (where the average snow water equivalent (SWE) exceeds 5 mm in the historical period of 1995-2014) under SSP126 and SSP585. For each panel, the solid and dotted lines represent future simulations with and without future LAP change, with respect to the subscript of LAP_{future} and LAP_{hist} seen in the legend. The background shading represents the standard deviation of RF based on the ELM simulations with different model configurations. Note that the p values from the Mann-Kendall (MK) test of statistical significance of the temporal trends from 2015-2100 for all the time series are smaller than 0.05, thus not shown inside each panel.

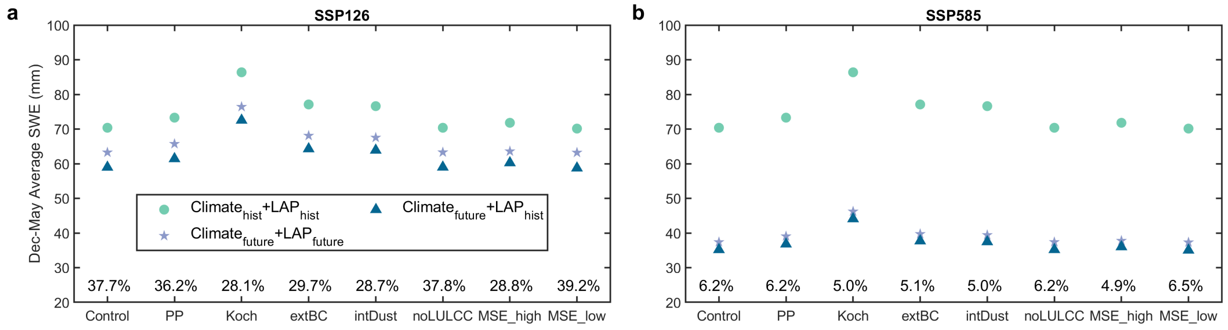

Figure S11| Historical and future average snow water equivalent (SWE) from December to May over the snow-covered Tibetan Plateau (TP) regions (where the average SWE exceeds 5 mm in the historical period of 1995-2014) under different model configurations: **(a)** SSP126 and **(b)** SSP585. For each panel, $Climate_{hist}$+LAP_{hist} represents the historical (1995-2014) simulations with historical light-absorbing particles (LAP) depositions, while $Climate_{future}$+LAP_{future} and $Climate_{future}$+LAP_{hist} represent future (2081-2100) simulations with and without a future change of LAP depositions, respectively. The $Climate_{future}$+LAP_{hist} simulations used the historical average LAP depositions from 1995-2014. The horizontal axis labels represent different model configurations (see **Methods**), where Control has the ELM default settings and the others represent major adjustments made from the Control case. Specifically, PP assumes that the terrain is flat and neglects topographic effects on solar radiation; Koch assumes a non-spherical snow grain shape (Koch snowflake); extBC assumes external mixing between hydrophilic BC and snow grains; intDust assumes internal mixing between dust and snow grains; noLULCC has no land use and land cover change; MSE_high assumes high melt-water scavenging efficiency (MSE = 2, much higher than the default value of 0.2) of hydrophilic BC; and MSE_low assumes a low MSE (0.02) of hydrophilic BC. In **(a,b)**, the contribution (δ_{LAP}) of future LAP change that mitigates snowpack loss under each ELM configuration is noted as a percentage and is calculated as the ratio of the SWE difference (ΔSWE_{LAP}) between $Climate_{future}$+LAP_{future} and $Climate_{future}$+LAP_{hist} to the SWE difference ($\Delta SWE_{Climate}$) between $Climate_{hist}$+LAP_{hist} and $Climate_{future}$+LAP_{future}.

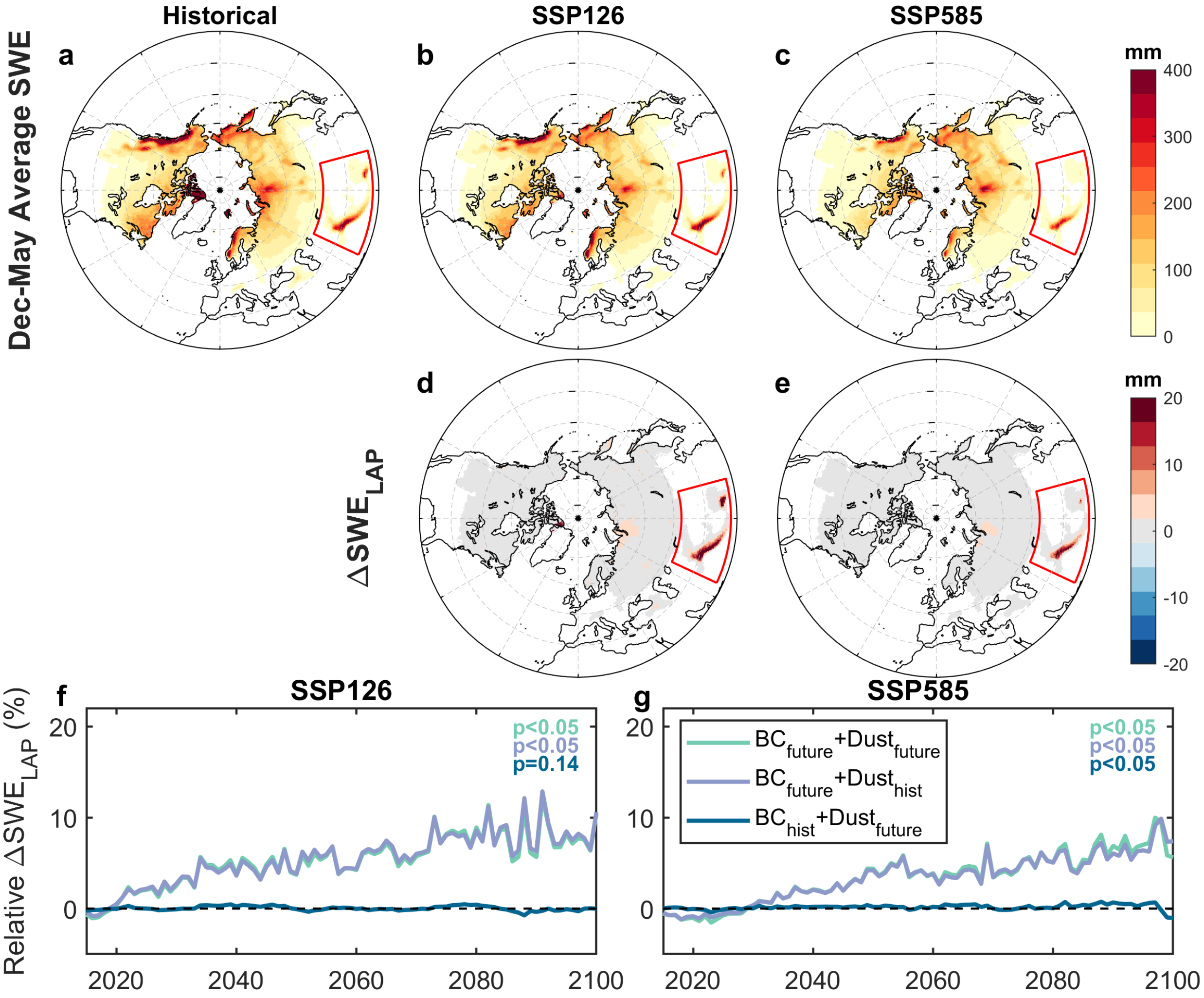

Figure S12| Future average snow water equivalent (SWE) from December to May and contributions of light-absorbing particles (LAP) change to future SWE. a Historical (1995-2014) and **b,c** future (2081-2100) spatial patterns of average SWE under SSP126 and SSP585. **d,e** The differences (ΔSWE_{LAP}) of future (2081-2100) SWEs with and without LAP change under SSP126 and SSP585. **f,g** Time series of relative ΔSWE_{LAP} (calculated as the ratio of ΔSWE_{LAP} to projected SWE without LAP change) over snow-covered regions (where the average SWE exceeds 5 mm in the historical period) in the Tibetan Plateau (TP). In (**a-e**), grids with an average SWE smaller than 5 mm in the historical period are masked. In (**f,g**), BC_{future}+$Dust_{future}$, BC_{future}+$Dust_{hist}$, and BC_{hist}+$Dust_{future}$ represent different combinations of BC and dust depositions, where the subscripts of future and hist represent future and historical average depositions, respectively. The p values from the Mann-Kendall (MK) test of statistical significance of the temporal trends from 2015-2100 are shown inside each panel.

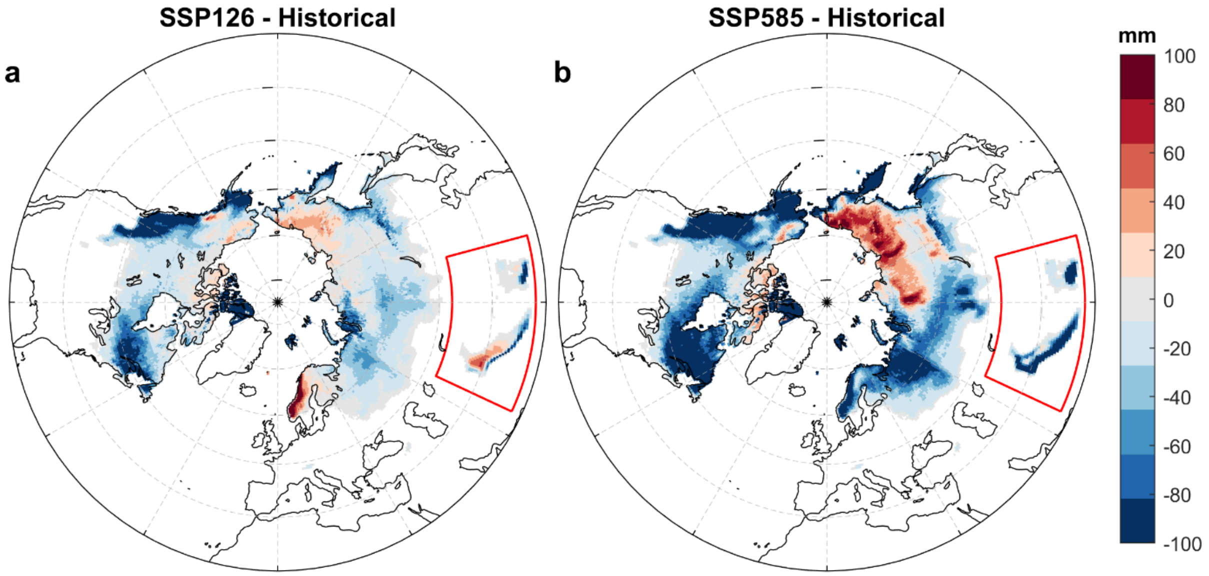

Figure S13| Spatial differences between future (2081-2100) and historical (1995-2014) snow water equivalent (SWE) **in April under (a) SSP126 and (b) SSP585.** Grids where the average SWE exceeds 5 mm in the historical period of 1995-2014 are masked**.**

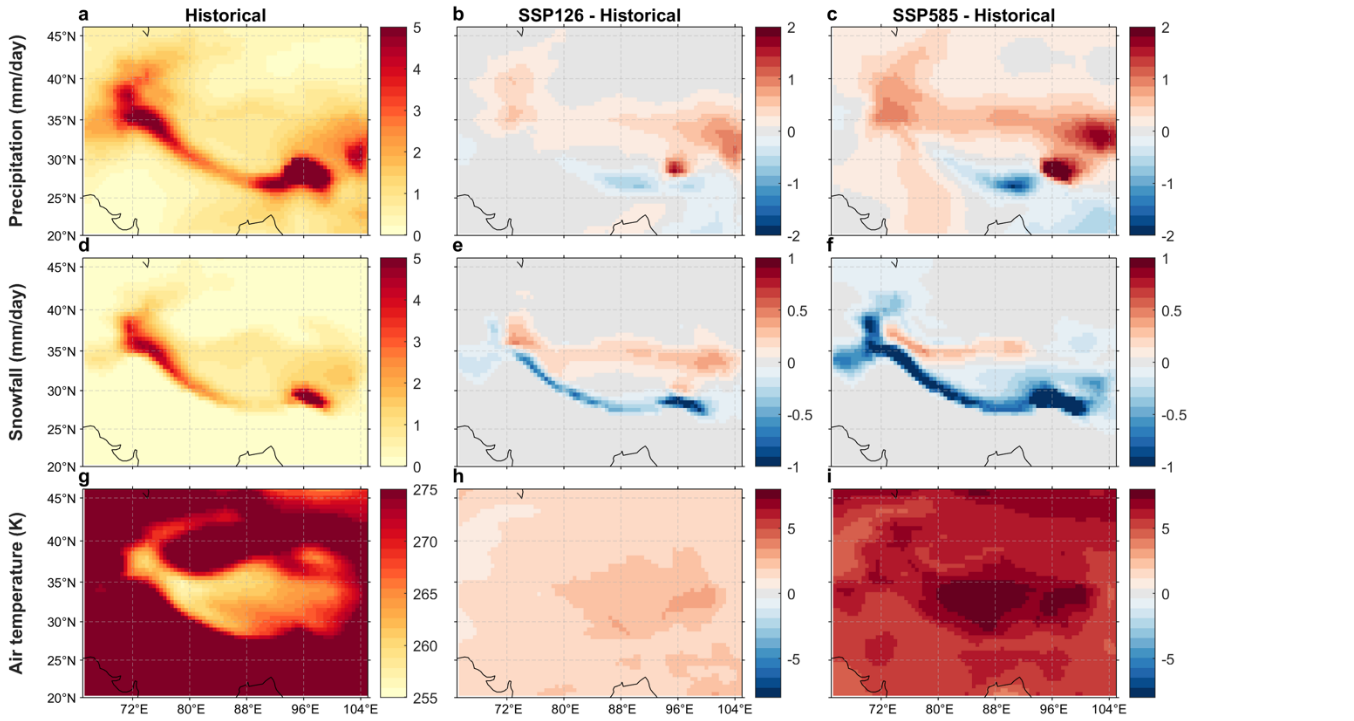

Figure S14| Spatial patterns of historical and future precipitation, snowfall and air temperature over the Tibetan Plateau (TP). a,d,g Historical (1995-2014) spatial patterns of climate conditions. **b,c,e,f,h,i** The difference between future (2081-2100) and historical climate conditions under SSP126 and SSP585. Historical and future climate conditions are calculated based on CESM outputs from December to May.

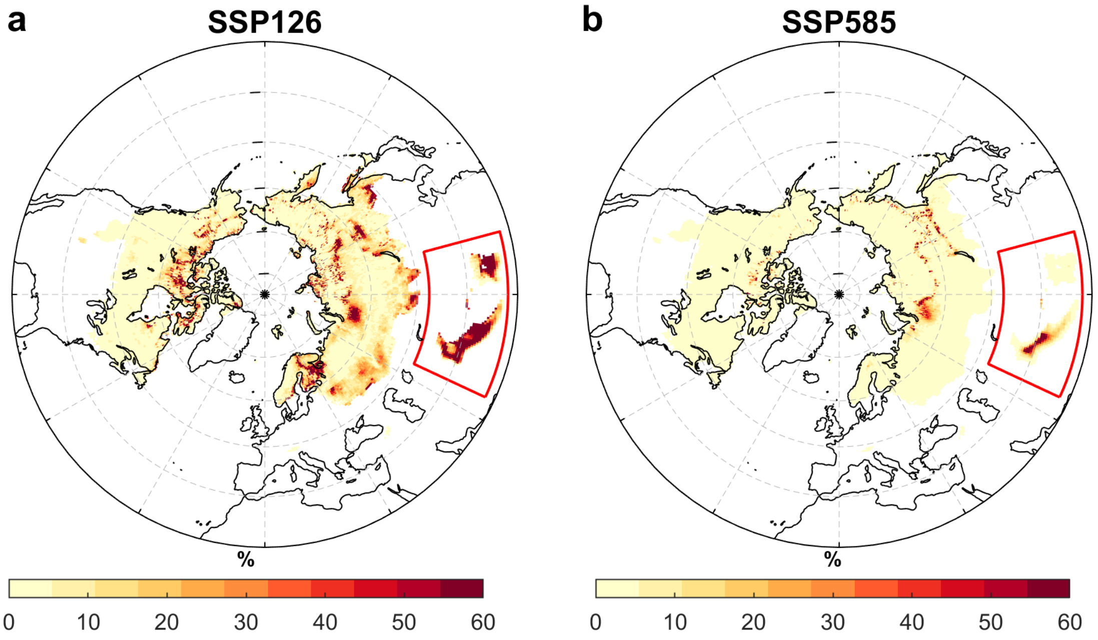

Figure S15| Spatial distributions of the contribution (δ_{LAP}, see Methods for its definition) of future light-absorbing particles (LAP) change to mitigating snowpack loss under (a) SSP126 and (b) SSP585. Grids where the average snow water equivalent (SWE) exceeds 5 mm in the historical period of 1995-2014 are masked.

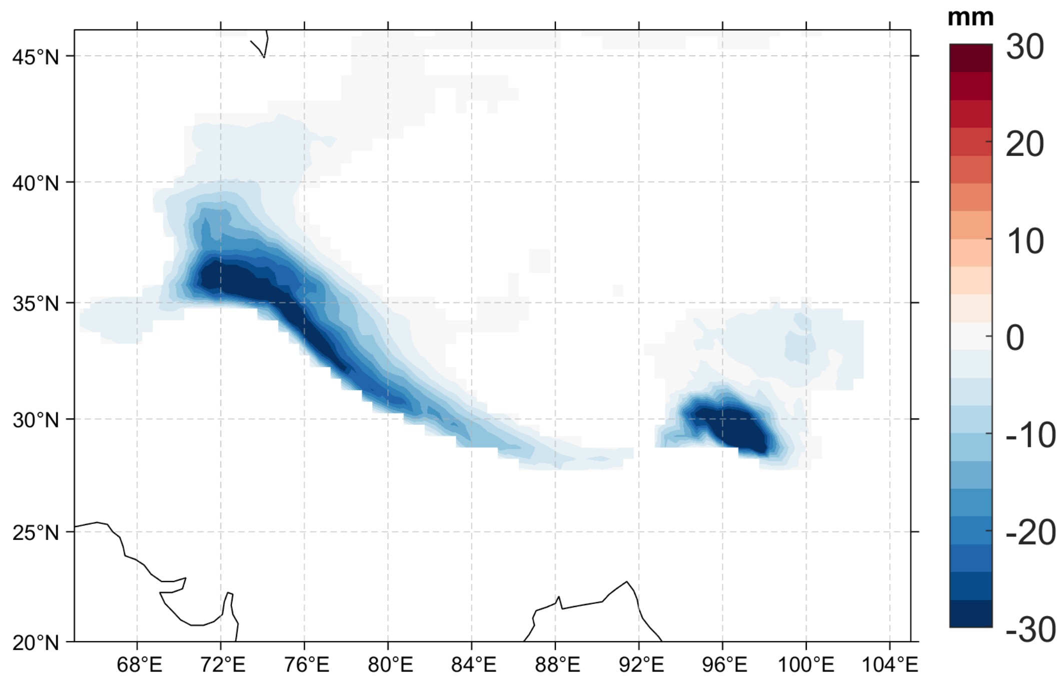

Figure S16| The black carbon (BC)-induced reduction of historical (1995-2014) average snow water equivalent (SWE) from December to May over the Tibetan Plateau (TP). The SWE reduction is calculated based on ELM historical simulations with and without BC deposition. The grids where the average SWE during December to May is smaller than 5 mm are masked.

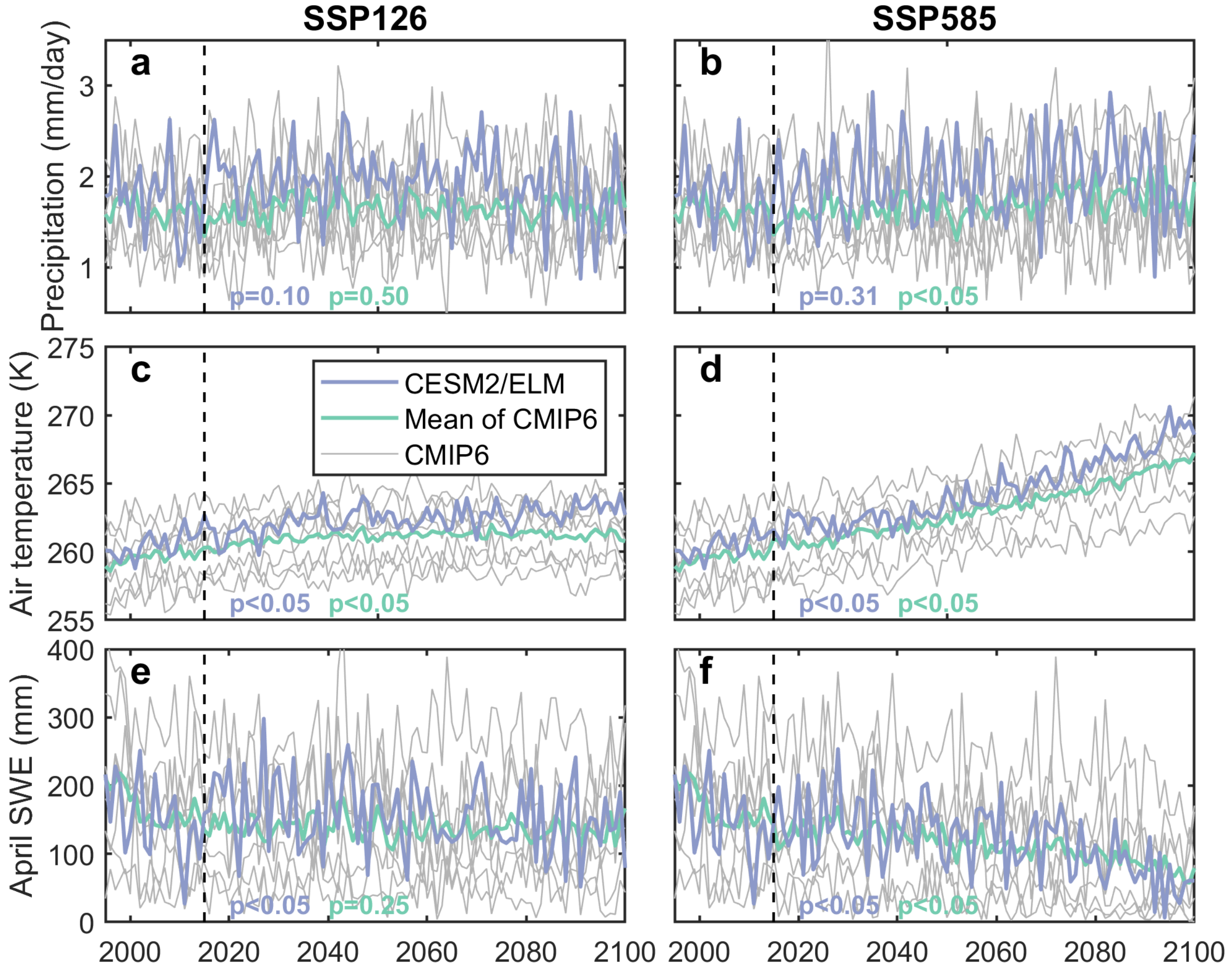

Figure S17| Time Series of (a,b) winter precipitation, (c,d) winter air temperature, and (e,f) April snow water equivalent (SWE) **over snow-covered Tibetan Plateau (TP) regions (where the average SWE exceeds 5 mm in the historical period of 1995-2014) under (a,c,e)SSP126 and (b,d,f) SSP585.** For each panel, the legend 'CESM2/ELM' represents either CESM2 data (precipitation or air temperature) or ELM simulated SWE; 'Mean of CMIP6' represents the ensemble mean of the seven CMIP6 models (Table S1) used in the study; 'CMIP6' represents each CMIP6 model used in the study; and the vertical dashed line indicates the year 2015 when the SSP scenarios start. The p values from the Mann-Kendall (MK) test of statistical significance of the temporal trends from 2015-2100 for CESM2/ELM and Mean of CMIP6 are shown inside each panel.

Table S1| Historical and future surface radiative forcings (RFs, Wm^{-2}) from December to May induced by black carbon (BC), dust, and BC+dust (i.e., the sum of BC and dust) over the snow-covered regions (where the average snow water equivalent (SWE) exceeds 5 mm in the historical period of 1995-2014) in the Northern Hemisphere (NH) **and Tibetan Plateau (TP) in ELM control simulations.**

LAP	NH			TP		
	Historical	SSP126	SSP585	Historical	SSP126	SSP585
BC	0.79	0.13	0.17	3.16	0.57	0.61
Dust	0.37	0.49	0.28	1.24	1.58	0.69
BC+dust	1.3	0.65	0.49	5.13	2.39	1.47

Table S2| Details of seven Earth system models in the CMIP6 experiments used in the study.

Model Name	Institute/Center	Original spatial resolution (longitude × latitude)	Reference
CanESM5	Canadian Centre for Climate Modelling and Analysis, Canada	2.8° × 2.8°	Swart et al.[2]
CESM2	National Center for Atmospheric Research, USA	1.25° × 0.94°	Danabasoglu et al.[3]
GFDL-ESM4	Geophysical Fluid Dynamics Laboratory, USA	1.25° × 1.0°	Held et al.[4]
INM-CM5-0	Institute of Numerical Mathematics of the Russian Academy of Sciences, Russia	2.0° × 1.5°	Volodin et al.[5]
MIROC6	Japanese modeling community, Japan	1.4° × 1.4°	Tatebe et al.[6]
MRI-ESM2-0	Meteorological Research Institute, Japan	1.9° × 1.9°	Yukimoto et al.[7]
NorESM2-LM	Norwegian Climate Center, Norway	2.5° × 1.9°	Seland et al.[8]

Table S3| ELM-based experiments with different model configurations. TOP and PP represent the shortwave radiation parameterizations with and without considering the topographic effects on solar radiation. MSE represents the melt-water scavenging efficiency of hydrophilic black carbon (BC).

**Case ID**	**Topographic effects on solar radiation**	**Snow grain shape**	**Mixing state of Hydrophilic-snow**	**Mixing state of dust-snow**	**Land use and land cover change**	**MSE**
Control	TOP	Spherical	Internal	External	Yes	0.2
PP	PP	Spherical	Internal	External	Yes	0.2
Koch	TOP	Koch Snowflake	Internal	External	Yes	0.2
extBC	TOP	Spherical	External	External	Yes	0.2
intDust	TOP	Spherical	External	Internal	Yes	0.2
noLULCC	TOP	Spherical	Internal	External	No	0.2
MSE_high	TOP	Spherical	Internal	External	Yes	2
MSE_low	TOP	Spherical	Internal	External	Yes	0.02

Supplementary References

1. Qian Y, Yasunari TJ, Doherty SJ, Flanner MG, Lau WKM, Ming J*, et al.* Light-absorbing particles in snow and ice: Measurement and modeling of climatic and hydrological impact. *Advances in Atmospheric Sciences* 2015, **32**(1)**:** 64-91.

2. Swart NC, Cole JNS, Kharin VV, Lazare M, Scinocca JF, Gillett NP*, et al.* The Canadian Earth System Model version 5 (CanESM5.0.3). *Geosci Model Dev* 2019, **12**(11)**:** 4823-4873.

3. Danabasoglu G, Lamarque J-F, Bacmeister J, Bailey DA, DuVivier AK, Edwards J*, et al.* The Community Earth System Model Version 2 (CESM2). *Journal of Advances in Modeling Earth Systems* 2020, **12**(2)**:** e2019MS001916.

4. Held IM, Guo H, Adcroft A, Dunne JP, Horowitz LW, Krasting J*, et al.* Structure and Performance of GFDL's CM4.0 Climate Model. *Journal of Advances in Modeling Earth Systems* 2019, **11**(11)**:** 3691-3727.

5. Volodin E, Gritsun A. Simulation of observed climate changes in 1850–2014 with climate model INM-CM5. *Earth Syst Dynam* 2018, **9**(4)**:** 1235-1242.

6. Tatebe H, Ogura T, Nitta T, Komuro Y, Ogochi K, Takemura T*, et al.* Description and basic evaluation of simulated mean state, internal variability, and climate sensitivity in MIROC6. *Geosci Model Dev* 2019, **12**(7)**:** 2727-2765.

7. Yukimoto S, Kawai H, Koshiro T, Oshima N, Yoshida K, Urakawa S*, et al.* The Meteorological Research Institute Earth System Model Version 2.0, MRI-ESM2.0: Description and Basic Evaluation of the Physical Component. *Journal of the Meteorological Society of Japan Ser II* 2019, **97**(5)**:** 931-965.

8. Seland Ø, Bentsen M, Olivié D, Toniazzo T, Gjermundsen A, Graff LS*, et al.* Overview of the Norwegian Earth System Model (NorESM2) and key climate response of CMIP6 DECK, historical, and scenario simulations. *Geosci Model Dev* 2020, **13**(12)**:** 6165-6200.
