# Supplementary material for: A cleaner snow future mitigates Northern Hemisphere snowpack loss from warming
Source: Nat Commun. 2023 Oct 2;14:6074. doi: 10.1038/s41467-023-41732-6 (PMC10545800; doi:10.1038/s41467-023-41732-6)
Supplement: Supplementary file 3 — Description of additional supplementary files [file 41467_2023_41732_MOESM3_ESM.pdf]

<u>Description of Additional Supplementary Files</u>

Supplementary Data 1: Summary of the historical field measurements of BC and dust concentrations in snow across the North Hemisphere, used in the study.
